# Electrochemical Sensors for Chloramphenicol: Advances in Food Safety and Environmental Monitoring

**DOI:** 10.3390/ph18091257

**Published:** 2025-08-24

**Authors:** Matiar M. R. Howlader, Wei-Ting Ting, Md Younus Ali

**Affiliations:** 1 Department of Electrical and Computer Engineering, McMaster University, 1280 Main Street West, Hamilton, ON L8S 4K1, Canada; 2 Department of Integrated Biomedical Engineering and Health Sciences, McMaster University, 1280 Main Street West, Hamilton, ON L8S 4K1, Canada; 3 Department of Chemical Engineering, National Taiwan University of Science and Technology, No. 43 Keelung Road Section 4, Taipei 106, Taiwan; wei-ting.ting@emse.fr; 4 Taiwan Building Technology Center, National Taiwan University of Science and Technology, No. 43 Keelung Road Section 4, Taipei 106, Taiwan; 5 Département BEL, Centre CMP, Mines Saint-Etienne, 880 Route de Mimet, F-13541 Gardanne, France; 6 Electronics and Communication Engineering Discipline, Khulna University, Sher-E-Bangla Road, Khulna 9208, Bangladesh

**Keywords:** electrochemical sensors, chloramphenicol, nanomaterials, biomarker detection, point-of-care diagnostic, antibiotic drugs

## Abstract

Excessive use of antibiotics can lead to antibiotic resistance, posing a significant threat to human health and the environment. Chloramphenicol (CAP), once widely used, has been banned in many regions for over 20 years due to its toxicity. Detecting CAP residues in food products is crucial for regulating safe use and preventing unnecessary antibiotic exposure. Electrochemical sensors are low-cost, sensitive, and easily detect CAP. This paper reviews recent research on electrochemical sensors for CAP detection, with a focus on the materials and fabrication techniques employed. The sensors are evaluated based on key performance parameters, including limit of detection, sensitivity, linear range, selectivity, and the ability to perform simultaneous detection. Specifically, we highlight the use of metal and carbon-based electrode modifications, including gold nanoparticles (AuNPs), nickel–cobalt (Ni-Co) hollow nano boxes, platinum–palladium (Pt-Pd), graphene (Gr), and covalent organic frameworks (COFs), as well as molecularly imprinted polymers (MIPs) such as polyaniline (PANI) and poly(*o*-phenylenediamine) (P(*o*-PD)). The mechanisms by which these modifications enhance CAP detection are discussed, including improved conductivity, increased surface-to-volume ratio, and enhanced binding site availability. The reviewed sensors demonstrated promising results, with some exhibiting high selectivity and sensitivity, and the effective detection of CAP in complex sample matrices. This review aims to support the development of next-generation sensors for antibiotic monitoring and contribute to global efforts to combat antibiotic resistance.

## 1. Introduction

Antibiotics are drugs widely used to treat bacterial infections and diseases in humans, animals, and plants [1,2]. While their application as growth promoters in livestock has been banned in regions, such as the United States (US) and the European Union (EU), they are still used—legally or illicitly—in many parts of the world. Antibiotics exert their effects by disrupting bacterial DNA/RNA replication, impairing cell wall synthesis, or interfering with metabolic processes, ultimately leading to bacterial cell death. However, the overuse and misuse of these drugs have accelerated the emergence of antibiotic-resistant bacteria—microorganisms that can withstand previously effective treatments [3]. This phenomenon, known as antimicrobial resistance (AMR), poses a global health crisis. By 2050, AMR is projected to cause up to 10 million deaths annually and force an estimated 28.3 million people into extreme poverty due to increased medical costs, which could exceed USD 300 billion globally [4]. Additionally, improper disposal of antibiotics contributes to environmental contamination, especially in aquatic ecosystems, where persistent residues exert selective pressure that fosters resistance. Tackling antibiotic resistance requires integrated efforts in healthcare, agriculture, and environmental management to mitigate its profound human and ecological consequences.

Among the various antibiotics subject to regulatory control, chloramphenicol (CAP) occupies a uniquely critical position due to its severe toxicological profile, idiosyncratic adverse effects, and zero-tolerance residue policies in many jurisdictions [5]. Even trace exposure has been associated with irreversible aplastic anemia, a condition with high mortality and no clear dose–response threshold, prompting regulatory limits set at or below the current analytical limit of detection at sub-µg/kg levels in the EU, US, and Australia [5,6]. These limits are among the most stringent for any veterinary drug residue, necessitating analytical tools with exceptionally high sensitivity and specificity. Furthermore, the physicochemical characteristics of CAP, including high polarity, poor volatility, and stability in environmental media, complicate its detection in complex matrices such as aquaculture water, soil, and processed food products [5]. These properties, combined with the ongoing illicit use of CAP in certain regions and its environmental persistence, make it both a public health concern and a technically challenging target for rapid detection platforms.

**Figure 1:**
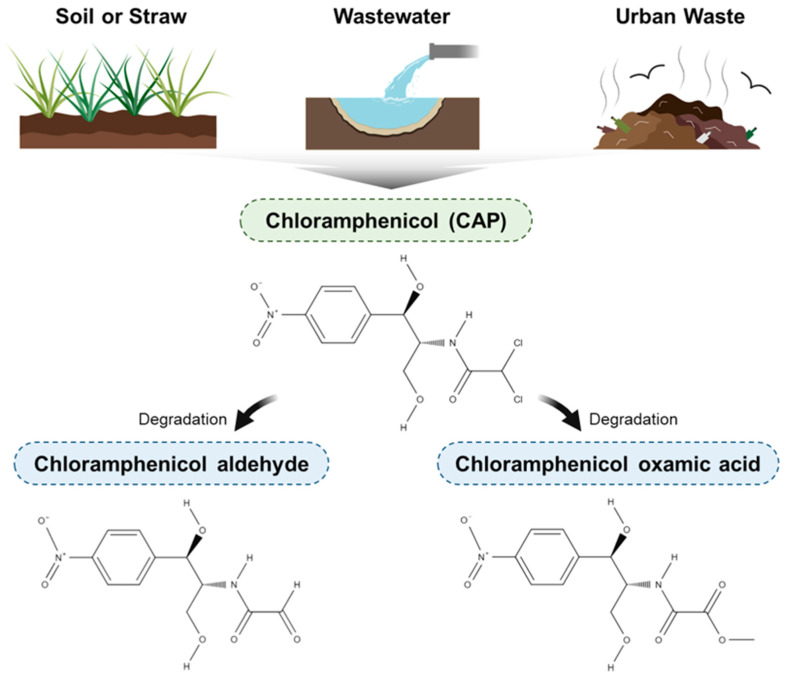
CAP alongside its major anthropogenic release pathways and chemical structures of CAP and its toxic degradation products.

CAP is a broad-spectrum antibiotic originally derived from the Gram-positive bacterium *Streptomyces venezuelae*. It exhibits potent activity against a range of Gram-negative and some Gram-positive bacteria by reversibly binding to the 50S subunit of the prokaryotic 70S ribosome, thereby inhibiting peptidyl transferase activity and blocking protein synthesis [7,8]. Despite its effectiveness, CAP has been banned or heavily restricted in numerous countries—including Canada, the US, the EU, Australia, and Japan—for use in human medicine, food, and food-producing animals due to its severe toxicological profile [5]. The primary health concerns associated with CAP include dose-dependent reversible bone marrow suppression, idiosyncratic aplastic anemia, blood dyscrasias, hepatotoxicity, neurotoxicity, and gray baby syndrome in neonates [9,10,11]. Aplastic anemia, in particular, is a rare but often fatal condition believed to result from a combination of genetic susceptibility and the formation of toxic nitroso metabolites. Table 1 summarizes the physiological and chemical mechanisms implicated in CAP-induced toxicities. Moreover, CAP is metabolized into reactive derivatives such as chloramphenicol aldehyde and chloramphenicol oxamic acid, which have been shown to damage hematopoietic cells in preclinical models [7]. The molecular structures of these metabolites are illustrated in Figure 1. Although banned for systemic administration, topical ophthalmic formulations (e.g., eye drops) remain in use due to minimal systemic absorption and a significantly lower risk of systemic toxicity when administered locally.

In addition to these topical applications, CAP is still employed in certain regions as a last-resort systemic antibiotic for severe infections, including typhoid fever caused by *Salmonella typhi*; bacterial meningitis due to *Haemophilus influenzae*, *Streptococcus pneumoniae*, and *Neisseria meningitidis*; anaerobic infections involving *Bacteroides* spp.; rickettsial diseases such as typhus and Q fever; and brucellosis [15,16]. These therapeutic applications, although limited to cases where safer alternatives are unavailable, contribute to CAP release into wastewater and soil via human excretion and hospital effluents, thereby representing additional environmental sources of contamination. According to the 2025 European Committee on Antimicrobial Susceptibility Testing (EUCAST) clinical breakpoint and guidance, CAP may be administered intravenously at a dosage of 2 g × 4 oral (total daily dose of 8 g) in the treatment of bacterial meningitis caused by susceptible strains [17]. Although such regimens are rarely adopted today, they underscore the continued clinical relevance of CAP under exceptional circumstances.

Environmental exposure to trace levels of CAP is common in agricultural settings, where animals may come into contact with CAP-contaminated soil, straw, or water. However, such passive exposure typically does not result in the accumulation of CAP residues in animal tissues at detectable levels (Figure 1) [18,19]. In contrast, the deliberate and illegal administration of CAP—particularly in livestock and aquaculture—can lead to significant residue accumulation in edible tissues, posing risks to consumer health due to CAP’s known toxic effects and broad-spectrum antibacterial activity. Consequently, the reliable detection of CAP residues in food products is essential for ensuring food safety, enforcing regulatory compliance, and minimizing unintentional antibiotic exposure in human populations.

Various analytical techniques have been developed for CAP detection, including immunoassays, chromatography (e.g., HPLC and GC-MS), and optical sensors. While these techniques offer high specificity and accuracy, they are often hindered by high operational costs, limited portability, complex sample preparation, and reduced applicability to field testing or routine screening. In contrast, electrochemical sensors have emerged as promising alternatives due to their high sensitivity, low cost, rapid response time, and ease of integration into portable platforms [20,21]. Recent advances focus on the development of electrochemical sensors using nanostructured materials and molecular recognition elements for the selective and practical detection of CAP in complex food and drug matrices, making these tools increasingly viable for real-world monitoring applications.

To date, only a limited number of reviews have focused specifically on electrochemical sensors for CAP detection. Dong et al. reviewed aptamer-based electrochemical sensors up to 2020; however, their analysis lacked discussion on the critical influence of electrode materials on sensor performance [22]. Another broader review addressed the electrochemical detection of the amphenicol antibiotics class but similarly overlooked the critical role of material composition in determining sensitivity, selectivity, and stability [23]. More recently, Abhishek et al. reviewed the use of advanced nanomaterials—such as graphene, metal oxide nanoparticles, and other functional nanostructures—for the electrochemical detection of CAP and furazolidone [24]. While this review provided useful insights into material-based strategies, it remained limited in scope with respect to fabrication techniques, detection contexts, and comprehensive performance benchmarking across studies.

Despite these contributions, a critical gap remains in the literature: a systematic review that integrates material composition, fabrication methodologies, and sensor performance for real-world CAP detection. Such a review is essential to synthesize the current state of research, identify persisting limitations, and inform the design of next-generation electrochemical sensing platforms. While AMR represents the broader context of this study, the focus of the present review is specifically on the detection of CAP residues, which serve as critical indicators of regulatory non-compliance and potential drivers of resistance development, rather than on the direct detection of resistance-conferring genetic traits. In this review, we critically evaluate recent advances in electrochemical sensors for CAP detection, with a focus on material-engineered interfaces and fabrication strategies. We assess their performance based on key analytical parameters, including limit of detection, sensitivity, linear dynamic range, selectivity, and potential for multiplexed or simultaneous analyte detection in food products and environmental waters.

## 2. Existing Diagnostic Tools

The use of the CAP antibiotic has been globally banned or strictly regulated in food-producing animals and human medicine due to its severe toxicological effects [5]. Consequently, multiple sensitive, specific, and robust analytical tools have been developed over the past three decades to detect CAP in food, pharmaceutical, and environmental samples. Many of these detection strategies are employed to improve result accuracy and minimize false positives and negatives.

Immunoassay detection methods are widely utilized for identifying specific analytes, including antibiotics such as CAP, due to their high sensitivity and specificity. These assays operate on the principle of competitive binding, wherein free CAP molecules in the sample compete with labeled CAP analogs for a limited number of antibody binding sites [25]. Among immunoassays, radioimmunoassay employs radioisotope-labeled compounds as tracers, providing excellent sensitivity but necessitating stringent handling protocols and specialized equipment due to radiological hazards. A more widely adopted alternative is the enzyme-linked immunosorbent assay (ELISA), which has become a standard technique for detecting the amphenicol class in food products and drugs. In a conventional ELISA format, an enzyme-conjugated antibody or antigen reacts with a chromogenic substrate, generating a measurable color change. This colorimetric signal is proportional to the analyte concentration and can be quantified using spectrophotometric techniques, offering a practical balance between sensitivity and operational simplicity. However, limitations such as matrix effects, cross-reactivity, and the need for laboratory infrastructure have prompted the development of more portable and field-deployable diagnostic alternatives.

Chromatography techniques are used to separate and identify chemical components in complex mixtures and are often employed in combination with immunoassays such as ELISA for CAP detection. Gas chromatography separates volatile compounds in the gas phase, while liquid chromatography separates components dissolved in a solvent. Gas chromatography is highly sensitive but can be tedious due to the need for analyte derivatization and potential interference from other compounds in the CAP peak [24,26]. Researchers have developed techniques using particle beam or thermospray interfaces to avoid derivatization, but these methods have a higher limit of detection (LOD). Liquid chromatography does not require derivatization, but it is slow and expensive, requiring UV-diode array detection to confirm the results and avoid false positives [26]. Despite their high sensitivity, the cost and complexity of the apparatus are notable disadvantages of chromatographic methods.

Optical sensors have gained significant attention due to their quick response times, high sensitivity, and potential for miniaturization and field deployment [27]. They are often modified with aptamers, nanomaterials, or enzymes to enhance selectivity and signal amplification. Among them, colorimetric aptasensors are the simplest techniques to use for point-of-care and on-site applications, as the presence of CAP is indicated by a visible color change, and no additional software or equipment is required. However, the precise concentration of CAP in the solution cannot be determined. Chemiluminescence is another type of simple optical sensing method that emits cold light as a result of a chemical reaction; the intensity of the emitted light is measured using a luminometer to assess the CAP concentration. A major drawback of optical sensors is their susceptibility to interference from stray light sources, which can compromise the accuracy and reliability of the results.

Electrochemical sensors have emerged as a highly promising platform for the detection of antibiotics such as CAP, owing to their excellent sensitivity, low cost, and suitability for miniaturization and field deployment [28,29]. A typical electrochemical sensor comprises three main electrodes: (1) a working electrode, which serves as the active surface for analyte recognition and electrochemical reaction; (2) a reference electrode, which maintains a stable and known potential; and (3) a counter (or auxiliary) electrode, which completes the electrical circuit and facilitates ion flow in the electrolyte. All electrodes must exhibit high conductivity, electrochemical stability, and compatibility with the sensing environment. Detection occurs at the working electrode, where the target analyte undergoes oxidation or reduction. The resulting electron transfer produces a measurable electrical signal, which is analyzed using techniques such as cyclic voltammetry (CV), differential pulse voltammetry (DPV), square-wave voltammetry (SWV), amperometry, linear sweep voltammetry (LSV), and electrochemical impedance spectroscopy (EIS). The presence of CAP is confirmed by observing a characteristic current peak or impedance change that is distinguishable from signals generated by background noise or interfering substances. Electrochemical sensors are particularly advantageous due to their high analytical performance, rapid response time, and potential for integration with portable devices. Moreover, their fabrication can be achieved using cost-effective materials and scalable methods, making them ideal candidates for PoC testing, food safety monitoring, and environmental surveillance.

## 3. Advances in CAP Detecting Electrochemical Sensors

### 3.1. Metal-Based Electrodes

#### 3.1.1. Gold—High Affinity

**Figure 2:**
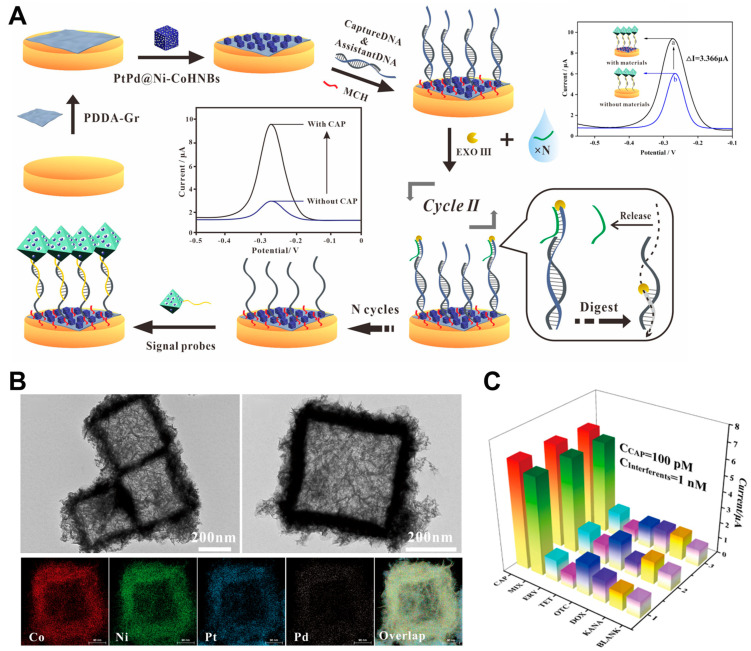
(**A**) Scheme of the construction of a dual-amplified aptasensor based on PDDA-functionalized graphene (PDDA-Gr) and PtPd@Ni-Co nanocubes. (**B**) Images of transmission electron microscopy (TEM) and energy-dispersive X-ray spectrometry (EDS) of PtPd@Ni-Co nanocubes showing uniform cubic morphology. (**C**) DPV responses of the aptasensor toward different samples, demonstrating the specificity of CAP detection. Adapted with permission from Ref. [30]. Copyright 2021 American Chemical Society.

Among the various types of metal electrodes, gold-based electrodes are widely used in electrochemical sensing due to their excellent electrical conductivity, chemical stability, and biocompatibility. Gold surfaces can be functionalized with aptamers, which are short DNA or RNA sequences that selectively bind to target analytes, thereby enabling the sensitive and specific detection of CAP. Aptamers enhance the specificity of gold electrodes (GEs) because of their high selectivity, low cost, and ease of synthesis. Wang et al. developed a modified GE by incorporating platinum–palladium (Pt-Pd) and nickel–cobalt (Ni-Co) hollow nanoboxes [30]. These nanostructures possess a high surface-to-volume ratio and a wrinkled morphology that facilitates efficient aptamer immobilization, as shown in Figure 2A,B. The platinum (Pt)-containing bimetallic nanostructures, particularly Pt-Pd, were selected to improve catalytic activity and electrical conductivity, since bimetallic systems generally perform better than single-metal counterparts. The resulting sensor exhibited high selectivity, showing no significant change in DPV peak current when exposed to other antibiotics at concentrations of 1 nM, while successfully detecting CAP at 100 pM (Figure 2C). Additionally, the sensor’s long-term stability was assessed over 14 days, and the initial current peak was reduced by 9%, which may have been a result of DNA denaturation.

**Figure 3:**
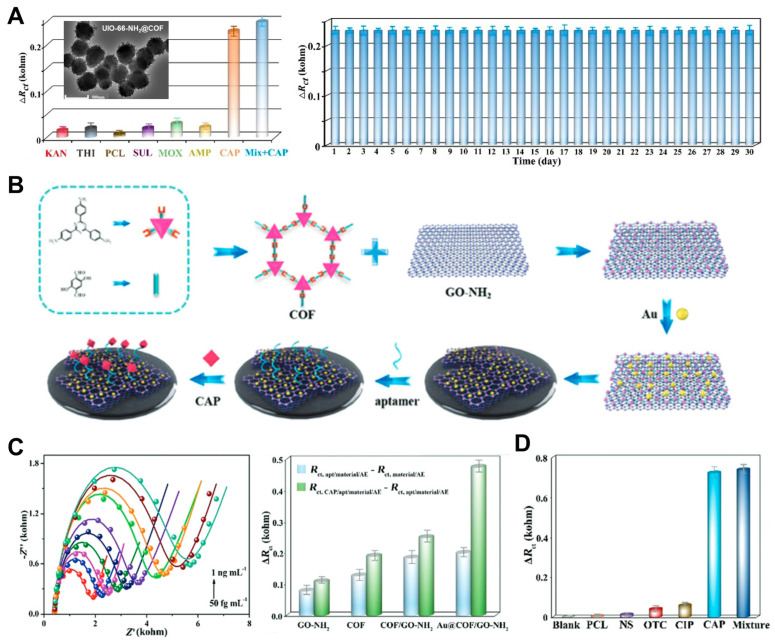
(**A**) Selectivity performance of the UIO-66-NH_2_@COF-based sensor against various antibiotic interferences and results of a 30-day long-term stability test. Adapted with permission from Ref. [31]. Copyright 2021 Elsevier. (**B**) Scheme of the functional Au@COF/GO-NH_2_-based aptamer sensor for CAP detection. (**C**) EIS analysis used to evaluate the sensitivity and (**D**) selectivity of the sensor. Adapted with permission from Ref. [32]. Copyright 2021 Royal Society of Chemistry.

An aptamer is a biorecognition element, an artificial DNA sequence generated through the process of systematic evolution of ligands by exponential enrichment (SELEX), designed for the selective detection of a target analyte. The aptasensor designed by Zhang et al. used UIO-66-NH_2_@COF, which has a retentive crystallinity and low weight density. Covalent organic framework (COF)-based aptasensors have mesoporous structures that provide excellent interactions with aptamers [31]. After being stored for 30 days, the sensor showed little to no change in its CAP response peak and had excellent selectivity toward CAP among other antibiotics (Figure 3A). To overcome the intrinsic limitations in the electrical conductivity of COFs, another aptasensor design integrated gold nanoparticles (AuNPs) and graphene oxide (GO), as depicted in Figure 3B [32]. The porous structure of AuNPs facilitates aptamer immobilization and promotes the formation of a uniform COF film. This COF layer is covalently anchored onto the GO-NH_2_ surface through a linking process between COF and GO-NH_2_. The analytical performance of the Au@COF/GO-NH_2_ aptasensor was assessed using CAP as the target analyte, revealing a well-defined linear response range (Figure 3C). Furthermore, the sensor exhibited excellent selectivity, as evidenced by negligible signal responses in the presence of structurally unrelated antibiotics at concentrations up to 10^7^-fold higher than that of CAP (Figure 3D). The composites were ultrasonically dispersed onto pre-treated GEs and then immersed in the aptamer solution by both research teams. Yan et al. designed an aptasensor using a GE modified only with a thiolated aptamer [33]. The selectivity of the sensor was tested using four other antibiotics at a 100 nM concentration, including CAP. The electrode showed sensitivity only toward CAP, but selectivity should be further tested with lower concentrations of CAP compared to the interferents for stronger evidence.

**Figure 4:**
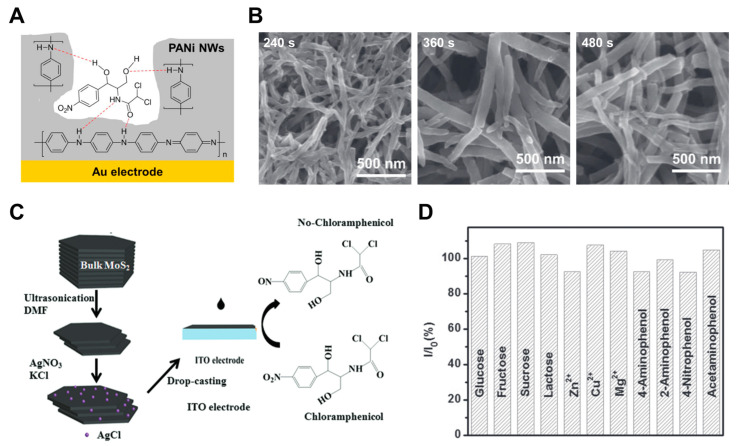
(**A**) Mechanism of hydrogen bonding between CAP and the recognition cavity of MIP-PANI, and (**B**) images of scanning electron microscopy (SEM) showing the growth of PANI NWs with increasing electrodeposition time. Adapted with permission from Ref. [34]. Copyright 2020 IOP Publishing. (**C**) The synthesis of AgCl/MoS_2_ composite on an ITO electrode for CAP detection, and (**D**) selectivity analysis of the sensor in the presence of various interfering species. Reproduced from Ref. [36]. Copyright 2019 American Scientific Publishers.

The final two GE-based sensors did not use aptamers but instead employed molecular imprinted polymers (MIPs), such as polyaniline (PANI), for electrochemical sensing. MIPs have higher surface-area-to-weight ratios and more effective binding sites, which can improve sensitivity [34,35]. These features often render MIPs more efficient than conventional biorecognition methods, such as enzymatic catalysis or antigen–antibody interactions. For instance, Chu et al. developed an MIP sensor based on one-dimensional PANI nanowires (NWs) with tunable electrical conductivity and long-term structural stability, as shown in Figure 4A [34]. The nanowires were directly electrodeposited onto a GE (Figure 4B); however, the study did not evaluate the sensor’s long-term stability or selectivity in the presence of interferents or complex matrices, limiting insight into its practical applicability.

Another sensor platform integrated bismuth tungstate (Bi_2_WO_4_) with MIP on a gold electrode through spin-coating. The device exhibited structural advantages attributed to the ultrathin morphology and the electromagnetic properties of Bi_2_WO_4_ [37]. However, it was not a purely electrochemical sensor in the strict sense, as it operated using a quartz crystal microbalance transduction mechanism, although the sensing interface involved electrochemical interactions. The sensor was evaluated with CAP and structurally similar antibiotics, including clindamycin, thiamphenicol, and florfenicol, all tested at equal concentrations of 0.5 mM. No significant signal interference was observed. Nevertheless, to comprehensively assess its selectivity and applicability in real-world scenarios, further studies are needed under conditions where CAP is present at trace levels in the presence of excess concentrations of other antibiotics.

#### 3.1.2. Other Metals—Highly Catalytic

Pt electrode-based sensors are widely used in electrochemical sensing due to their remarkable corrosion resistance and low reactivity. In a study by Zhao et al., Pt thin film microelectrodes were employed for their high precision, strong chemical stability, and excellent conductivity [38]. The Pt thin film electrodes were electropolymerized with the polymer of poly(*o*-phenylenediamine) (P(*o*-PD)), which enhances miniaturization and forms compact polymer films due to its high affinity, selectivity, and stability. To test selectivity, antibiotics with 10 times greater concentrations than that of CAP were used, but there was no effect on the CAP peak. However, the long-term stability of the sensor was not evaluated, so it is uncertain how long it can be used compared to other sensors. Another team also developed a Pt electrode-based sensor, but they did not provide the reasoning for their choice of materials or a limit of detection. Multiple ions and organic compounds were used as interferents, but no competing antibiotics were used to test for selectivity [39]. Some of the interferents had no effect on the CAP peak even at a 500 to 1 ratio, but some impacted the peak at ratios above 50. The sensitivity of the electrode to CAP could have been improved by modifying its surface with nanomaterials or aptamers.

One representative example of a metal-based sensor utilized indium tin oxide (ITO) as the working electrode [36]. While molybdenum disulfide (MoS_2_) offers a Gr-like layered structure advantageous for surface interactions, its practical sensitivity is limited by inherently low electrical conductivity. To overcome this drawback, silver chloride (AgCl) was incorporated with MoS_2_, and the resulting AgCl/MoS_2_ nanocomposite suspension was drop-cast onto the ITO surface (Figure 4C). Selectivity was evaluated against potential interferents, including common ions, saccharides, and antibiotics (Figure 4D). These species exhibited minimal interference with the CAP signal, demonstrating the sensor’s reasonable selectivity. Notably, the sensor retained 83.3% of its original current response after three weeks of storage, indicating moderate operational stability. However, residual signal suppression was observed, likely due to non-specific interactions between interferents (particularly saccharides and ions) and the electrode interface. This suggests that integrating more structurally stable or antifouling composite materials may further enhance selectivity and long-term reliability. A summary of representative metal-based electrochemical sensors for CAP detection is provided in Table 2.

### 3.2. Carbon-Based Electrodes

#### 3.2.1. Aptamer on Glassy Carbon Electrode (GCE)—Immobilize CAP

Roushani et al. have been working on developing a glassy carbon aptasensor with silver nanoparticles (AgNPs) for CAP detection [40]. The first sensor they developed used GO, a low-cost and stable material with a large surface area that can easily disperse in polar solvents to improve contact with catalytic active sites. Triethoxysilane (NH_2_-Si) was added to react with the functional groups of GO and enable aptamer immobilization by increasing the surface-to-volume ratio and speeding up electron transfer kinetics. The AgNP/NH_2_-Si composite was drop-casted onto a glassy carbon electrode (GCE). Antibiotics at concentrations 1000 times greater than CAP were used as interferents, and the sensor showed high selectivity toward CAP despite slight changes in the signal. The sensor retained 92% of its initial current peak after being stored for 2 weeks at 4 °C. The more recent sensor they developed used 3-aminomethyl pyridine-functionalized graphene oxide (3-ampy-RGO) instead of NH_2_-Si to immobilize the aptamer [41]. 3-ampy-RGO is an MIP with high selectivity but poor binding attachment, which can be improved by the aptamer’s high binding affinity. Instead of drop-casting, the electrode was covered with the composite materials, and the aptamer was dropped onto the electrode overnight. This newer sensor had a lower detection limit but only retained 89% of its initial current response after being stored for a week at 4 °C. Although the newer sensor has a higher sensitivity toward CAP, its long-term stability is significantly lower compared to the first sensor.

Another team developed an electrochemical sensor using a GCE immobilized with an aptamer, metal–organic frameworks (MOFs), and incorporated magnetic gold nanoparticles (MGNPs) [42]. To enhance signal amplification and target recycling, they further introduced a circular strand-replacement DNA polymerization strategy. This approach enabled the simultaneous detection of oxytetracycline and CAP with an exceptionally low LOD (0.033 pM) and exhibited excellent selectivity for CAP even in the presence of various antibiotics, proteins, and metal ions as potential interferents. In a related yet distinct strategy, another research group proposed an electrochemical sensor based on a dual-recognition mechanism integrating MIPs and aptamers for the highly sensitive and selective detection of CAP [43]. The synergistic use of MIPs and aptamers provided two complementary recognition sites, significantly enhancing the sensor’s specificity. To optimize electron transfer and signal transduction, conductive materials such as chitosan-functionalized multi-walled carbon nanotubes (CS-MWCNTs) and AuNPs were employed. CAP molecules selectively interacted with the imprinted cavities through hydrogen bonding and other non-covalent interactions, which impeded electron transfer and led to a decrease in current. A strong correlation between CAP concentration and current change enabled a quantitative analysis. After parameter optimization, the sensor achieved a broad linear detection range and a low LOD, demonstrating its potential for practical application in trace-level antibiotic residue monitoring.

#### 3.2.2. AuNPs on Carbon—Possesses Unique Structural and Chemical Properties

**Figure 5:**
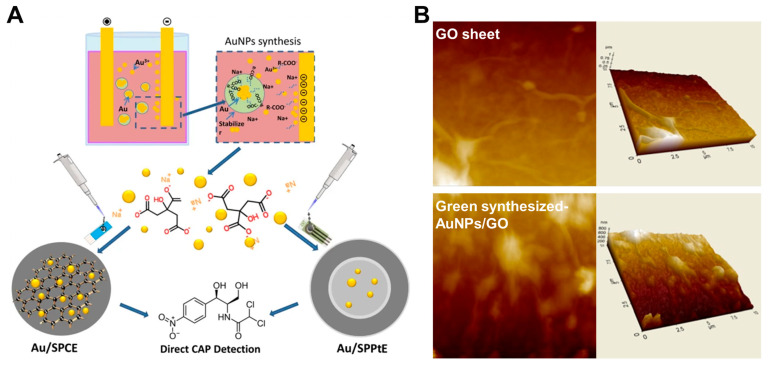
(**A**) Fabrication of electrodeposited AuNPs on screen-printed electrodes for the detection of CAP. Adapted with permission from Ref. [45]. Copyright 2022 Springer Nature. (**B**) 2D and 3D AFM images of GO and green-synthesized AuNPs on a GO sheet. Adapted with permission from Ref. [47]. Copyright 2016 Elsevier.

Gold nanomaterials are widely used in electrode modification due to their high chemical stability, high surface-to-volume ratio, and biological compatibility. A composite of gold nanoflowers and GO, which has high electrical conductivity, electrocatalytic activity, and mechanical stability, was drop-casted onto a screen-printed carbon electrode (SPCE) [44]. The sensor had a 4.11% deviation from its initial peak response after being stored for 14 days at 4 °C. In contrast, another team developed a comparatively less effective sensor by drop-casting AuNPs directly onto a different type of screen-printed electrode, as shown in Figure 5A [45]. While this electrode demonstrated good selectivity toward CAP in the presence of various ions, antibiotics, and organic interferents, it suffered from a significantly higher LOD. Moreover, the sensor was not evaluated using real samples nor assessed for long-term stability. The relatively low sensitivity of this platform could potentially be improved by integrating additional nanomaterials with higher electrical conductivity or synergistic catalytic properties.

Boron-doped diamond (BDD) electrodes are an uncommon option for a working electrode because there has been limited research on their use for the electrochemical sensing of CAP [46]. However, AuNPs were electrochemically deposited on BDD due to its chemical and physical stability and strong electrochemical behavior. The electrode was only tested in a buffer, not in real samples, so its potential for use in biofluids is uncertain. It was not evaluated against interferents or for long-term stability, so its full capabilities are unclear, apart from its limit of detection of 5 µM in the buffer solution.

Aside from their goal of creating an excellent CAP detection sensor, Karthik et al. focused on developing a green method for synthesizing AuNPs [47]. Gold salts were reduced using water-soluble phytochemicals extracted from the aqueous solution of *B. javanica Blume*, enabling an environmentally friendly and surfactant-free approach. The resulting green-synthesized AuNPs were composited with GO and drop-casted onto a GCE, as demonstrated in the atomic force microscopy (AFM) images in Figure 5B. These 2D and 3D AFM images clearly reveal the uniform distribution of AuNPs on the GO sheet, highlighting the nanoscale topography and surface roughness crucial for enhancing electron transfer and surface interaction with CAP. Selectivity was evaluated using ions (Ca, Cu, Mn, and Zn) and antibiotics at 500 times the concentration of CAP. 4-nitrophenol and 4-nitrobenzene had a response of 11–13% of the CAP signal, but the sensor was stable, retaining 92.15% of its initial current response after 15 days of storage.

In a related study, another research group developed a CAP sensor by modifying a GCE with a composite of gold nanoparticles and nitrogen-doped graphene (N-Gr) [48]. Gr, known for its large surface area and excellent electrical conductivity, benefits further from nitrogen doping, which introduces defect sites and enhances bonding capability through nitrogen’s lone pair electrons. This sensor was challenged with common interferents such as glucose, uric acid, ascorbic acid, and several antibiotics. While most species did not interfere with the CAP signal, compounds such as p-nitrophenol and p-nitrotoluene produced significant overlapping signals that hindered selectivity. Additionally, the sensor was tested using CAP eye drop formulations. However, considering that the primary application of electrochemical CAP sensors lies in monitoring environmental or food contamination (e.g., in water, soil, and aquaculture), the relevance of pharmaceutical formulations as test matrices is limited.

#### 3.2.3. Aptamer on Graphite—Selective to CAP

Rosy et al. designed an edge plane pyrolytic graphite (EPPG) sensor with poly(1,5-diaminonapthalene) (p(1,5-DAN)) and then dipped it in glutaraldehyde before drop-casting it with the aptamer [49]. 1,5-DAN is a polymer that exploits the cross-linking ability of glutaraldehyde, which can immobilize proteins and polymers containing free amino acids, thus immobilizing the aptamer onto the electrode surface. Cross-linking also strengthens the bonds, producing a more stable sensor. This sensor retained about 98% of its initial current response after 30 consecutive days of storage and is one of the most stable sensors reviewed in this review. The specificity of the sensor was studied using 50 µM of kanamycin and tetracycline as interferents. The interferents had no impact on the CAP current peak when the CAP concentration was 100 fM. Yadav et al.’s EPPG sensor was developed using poly-(4-amino-3-hydroxynaphthalene sulfonic acid) (p-AHNSA) as the polymer for aptamer immobilization [50]. After electropolymerization, the aptamer was spin-coated onto the electrode. The sensor has strong long-term stability, retaining 97% of its initial response after being stored for 30 days in a solution with a pH of 7.2. Other antibiotics, such as kanamycin and tetracycline, were used as interferents, with all antibiotics including CAP having a concentration of 100.0 nM, but there was no impact on the CAP signal.

#### 3.2.4. Carbon on Carbon—Highly Specific Surface Area

MWCNTs are widely utilized in sensor design due to their high surface area, excellent electrical conductivity, and ease of functionalization. In one approach, an MWCNT suspension was drop-casted onto a GCE, followed by the electrodeposition of copper nanodendrites (CuNDs) to further enhance surface reactivity and electron transfer efficiency [51]. To improve molecular selectivity, surface-imprinted polymers were employed, leveraging their enhanced binding capacity and kinetics when coupled with nanostructured materials like MWCNTs. The resulting MWCNTs@MIP composite was subsequently applied onto a GCE modified with ordered mesoporous carbon (CMK-3) and three-dimensional porous reduced graphene (P-r-Gr) [52]. Selectivity was assessed using organic compounds such as glucose, uric acid, ascorbic acid, and glutamic acid at concentrations 100 times greater than that of CAP, instead of using antibiotics as interferents. The selected compounds had no impact on the CAP peak. The sensor was also stable with 95.1% of its initial current response after being stored at room temperature for 20 days.

**Figure 6:**
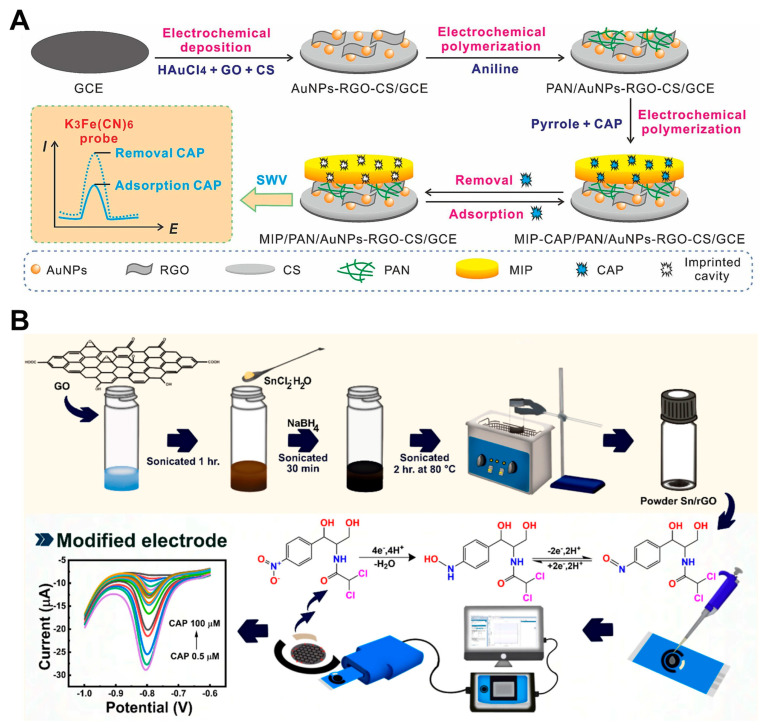
(**A**) Fabrication process of hybrid nanomaterial-based electrodes for CAP detection using a redox probe. Adapted with permission from Ref. [53]. Copyright 2025 Elsevier. (**B**) Preparation strategy of a hybrid nanostructure on SPCE for the electrochemical detection of CAP. Adapted with permission from Ref. [29]. Copyright 2024 American Chemical Society.

Building on the MWCNT@MIP framework, a recent study introduced an MIP-based electrochemical sensor integrating PANI, AuNPs, rGO, and chitosan (MIP/PANI/AuNPs-rGO-CS) on a GCE, as shown in Figure 6A [53]. This hybrid system combined high conductivity with abundant recognition sites, enhancing both signal amplification and molecular selectivity. Using potassium ferricyanide as a redox probe, the sensor achieved a low detection limit of 63 pM across dual linear ranges (1.0 nM to 8.0 nM and 10 nM to 200 nM). It also exhibited rapid adsorption/desorption (4/3 min), excellent stability, and high selectivity in complex samples such as milk and egg. Compared to MWCNT-based designs, this approach demonstrates a shift toward multifunctional nanocomposites that unify sensitivity, specificity, and practical applicability in real-sample analysis.

To explore alternative nanocarbon architectures, single-walled carbon nanohorns (SWCNHs) were also investigated. These materials possess similar advantageous properties to MWCNTs but offer enhanced chemical stability and electrical conductivity due to their unique conical structure and aggregated nanohorn morphology [54]. A GCE was modified with a carboxylated-SWCNH (SWCNHs-COOH) suspension; however, despite the intrinsic advantages of SWCNHs, the resulting sensor did not achieve an LOD compared to the MWCNT-based system. This outcome suggests that while SWCNHs have superior conductive potential, additional structural or material enhancements are needed. Indeed, such modifications could further increase the electrode surface area and active sites, potentially improving sensitivity and analytical performance.

Reduced graphene oxide (rGO) has been widely employed in sensors due to its fast electron transfer kinetics, high surface area, and excellent electrical conductivity. In a study by Zhang et al., three-dimensional reduced graphene oxide (3D-rGO) was utilized instead of 2D rGO to enhance the surface area [55]. The resulting electrode showed no alteration in its initial current peak, even after being stored for 30 days. Only organic compounds found in the body, such as glucose, cysteine, and uric acid, were used as interferents with concentrations ranging from 0.5 to 10 mM, while the concentration of the CAP was 0.5 µM. These compounds had an impact of less than 5% on the CAP signal. In comparison, another group utilized two-dimensional rGO on a GCE, which led to a higher LOD [56]. However, selectivity and storage stability were not assessed, and both studies omitted details on the method of rGO deposition, which could affect reproducibility.

To enhance the rGO performance, heteroatom doping was explored. Chlorine-doped rGO (Cl-rGO) was coated on GCEs, showing high selectivity against 10 mM concentrations of various ions and antibiotics, with no observable interference [57]. Nonetheless, all rGO-based sensors exhibited a relatively large LOD, suggesting that rGO alone may be insufficient for ultrasensitive CAP detection. To overcome these limitations, a more recent approach employed tin/reduced graphene oxide-modified (Sn/rGO) SPCEs for CAP sensing, as shown in Figure 6B [29]. This hybrid structure significantly improved electron transfer, increased electrocatalytic activity, and reduced fouling. The resulting sensor achieved a lower LOD and superior sensitivity compared to conventional rGO/GCE configurations. Furthermore, the use of SPCEs offered advantages in portability and reproducibility, making them suitable for on-site environmental monitoring.

Among graphene-based platforms, graphene nanoflakes (GNFs) have attracted attention due to their abundant edge defects, which enhance electron transfer. A sensor fabricated with GNFs was capable of simultaneously detecting CAP and metronidazole at low concentrations [58]. The system exhibited excellent long-term stability, maintaining 99.2% and 98.5% of its current response after two and four weeks, respectively. Only minor suppression of the CAP peak was observed when 0.01 mM of other antibiotics was introduced.

Beyond graphene, mesoporous carbon materials have also been studied. CMK-3, despite its high surface area and excellent stability, suffers from poor dispersibility with GCEs. This was addressed by coating CMK-3 with polydopamine (PDA), followed by electropolymerization with β-cyclodextrin (β-CD) [59]. The hydroxyl groups of the CAP formed hydrogen bonds with β-CD, enhancing the sensing signal. This composite electrode retained 86% of its initial signal after 30 days. The sensor demonstrated high selectivity, with less than 7% signal deviation in the presence of antibiotics and common organic interferents at equimolar concentrations.

Carbon black (CB)-incorporated polymers are one of the known commercial conductive filaments [60]. The CB-integrated polylactic acid (PLA) filaments were inexpensively extruded using 3D pens. Selectivity was assessed using interferents such as sodium citrate and uric acid, which can be found in milk. The interferents caused a drop of less than 15% in the CAP peak when their and CAP’s concentrations were the same. The electrode’s long-term stability was not assessed.

Lastly, pencil graphite electrodes (PGEs), known for their low cost, good stability, and consistent structure, were evaluated for CAP detection [61]. Despite their simplicity, the unmodified PGEs exhibited relatively high LODs. Furthermore, they were neither tested in real samples nor challenged with interfering species, limiting their practical assessment. Surface modification with noble metals or aptamers may substantially enhance their sensitivity and applicability. Representative carbon-based electrochemical sensors for CAP detection are summarized in Table 3.

### 3.3. Other Hybrid Electrodes

**Figure 7:**
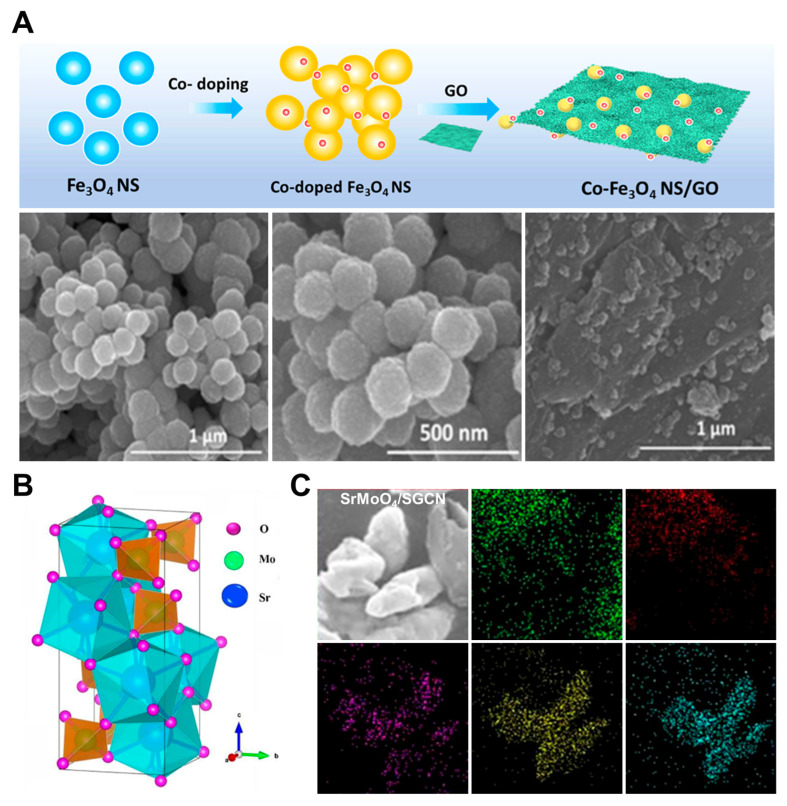
(**A**) Step-by-step synthesis of Co-Fe_3_O_4_ NS/GO composites and their corresponding SEM images. Adapted with permission from Ref. [64]. Copyright 2021 American Chemical Society. (**B**) Crystal structure of SrMoO_4_, along with (**C**) SEM and EDS images of the SrMoO_4_/SGCN composite. Adapted with permission from Ref. [65]. Copyright 2021 Elsevier.

Iron oxide (Fe_3_O_4_) nanoparticles can facilitate fast electron transfer and are easy to prepare, but they have low conductivity and are not very stable [62,63,64]. Bai et al. used Fe_3_O_4_ to modify a carbon fiber microelectrode (CFME), which has high fracture toughness and conductivity [62]. However, the sensor had poor long-term stability, maintaining only 83% of its initial current response after 10 days of storage at room temperature. When assessed for selectivity using other antibiotics as interferents with concentrations ten times higher than CAP, the signal was not affected. Another team electrochemically deposited Fe_3_O_4_ onto a GCE [63]. This electrode had a higher LOD than the first, and its long-term stability was not assessed. When tested for selectivity using ions with concentrations 80 times higher than CAP and antibiotics with concentrations five times higher, there was no impact on the CAP signal. The electrochemical performance of Fe_3_O_4_ can be improved further by doping with metal ions. Nehru et al. doped Fe_3_O_4_ with cobalt, forming a cobalt-doped Fe_3_O_4_ nanosphere deposited on graphene oxide (Co-Fe_3_O_4_ NS/GO) that was drop-cast onto a GCE, as shown in Figure 7A [64]. The signal had less than 5% error when assessed for selectivity using metal ions and biomolecules as interferents.

Cobalt oxide (Co_3_O_4_) is a low-cost, highly stable, and easily available material with good electrocatalytic properties and has the potential for oxygen reduction [66,67]. One team doped Co_3_O_4_ with holmium (Ho) to improve its electrochemical performance and placed the composite on a GCE. Uric acid, rutin, tyrosine, and glucose were some of the selected interferents used with concentrations 10 times greater than CAP to assess selectivity, and they affected the signal by up to around 6% of the original peak [66]. This sensor was tested in eye drops, human urine, and blood sera, which is not very applicable in real life. CAP is usually consumed safely under the guidance of a medical practitioner, so it is important to develop sensors that can detect CAP in food, water, and soil to protect people from unnecessary consumption. Another team drop-casted a Co_3_O_4_@rGO suspension on a GCE due to GO’s amazing electrochemical properties and its oxygen functional groups that allow it to bond easily with other compounds [67]. Although this sensor had a larger LOD than the previous one, it was highly selective toward CAP when interferents such as uric acid, cysteine, and glutathione, with the same concentrations as CAP, were used to check for interference. This electrode maintained 91.7% of its initial current response after being stored for 20 days.

Strontium (Sr) and its derivatives have emerged as a promising biocompatible material, characterized by a high surface-area-to-volume ratio and remarkable electrocatalytic activity. Its nanostructures, exhibiting an almond-like morphology, as presented in Figure 7B, contribute significantly to its electrochemical performance by offering abundant active sites and favorable charge transfer dynamics [65]. To improve catalytic functionality, graphitic carbon nitride (GCN), a chemically stable and biocompatible material, was doped with sulfur to form sulfur-doped graphitic carbon nitride (SGCN). This modification enhances both ionic and electronic conductivity and alters the electronic structure of the tri-s-triazine rings in GCN. As a result, the catalytic activity of GCN is significantly improved due to more efficient charge transport and an increased number of reactive sites [65]. A composite material was subsequently constructed by embedding strontium molybdate (SrMoO_4_) into the SGCN matrix and integrating the resulting hybrid onto a GCE. Figure 7C confirms that the almond-shaped SrMoO_4_ particles are successfully conjugated with the SGCN network. This composite electrode maintained 95.63% of its initial peak current after 30 days of storage and exhibited high repeatability with a relative standard deviation of 3.62 percent, indicating excellent stability and reproducibility. In a related study, another research team developed a nanocomposite consisting of strontium-doped zinc oxide (Sr-ZnO) combined with rGO. This composite demonstrated strong interfacial binding, high tolerance to variations in isoelectric point, and was amenable to further chemical modification [68]. When applied to an SPCE by drop-casting, the Sr-ZnO/rGO composite retained 88% of its original electrochemical response after 20 days of storage at 4 degrees Celsius. In addition, common electrochemical interferents such as 4-aminophenol, nitrophenol, 4-acetamidophenol, and 0.1 M CAP induced only minimal shifts in the CAP detection peak, thereby affirming the sensor’s selectivity and operational robustness.

Copper (Cu) nanoparticles have high conductivity and are cost-effective. Cuprous oxide (Cu_2_O) and cupric oxide (CuO) nanoparticles are even better conductors and have been successful in detecting antibiotics [69]. Cu-MoS_2_ was also tested because MoS_2_ nanosheets have a large surface area and excellent chemical stability [69]. All of these nanocomposites and copper nanoparticles were evaluated for their potential in CAP detection and were drop-casted onto individual working screen-printed electrodes. The electrode maintained 83.4–89.6% of its initial current peak after being stored for 30 days at room temperature. Glucose, ascorbic acid, 4-nitrophenol, and amoxicillin were some of the interferents used to test selectivity. At concentrations ten times greater than CAP, amoxicillin showed 17–23% of the CAP current response. This might be because amoxicillin contains copper, which has a higher affinity for copper than the atoms on CAP. Spinel oxides (AB_2_O_4_) have low limits of detection, good stability, and excellent electrocatalytic efficiency [70]. Copper cobaltite (CCO) and copper ferrite (CFO) have valence electrons in the p and d orbitals, making it easy to bind to analytes and improve conductivity. CCO and CFO suspensions mixed with GO were dripped onto individual electrodes. A total of 0.5 mM of interferents, including ascorbic acid, glucose, and thiourea, as well as 30 µM of CAP, were used to assess selectivity, but the interferents had no impact on the peak.

Manganese tungstate (MnWO_4_) has low toxicity and good electrochemical properties for metal coordination and can be easily synthesized. MnWO_4_ was combined with graphitic carbon nitride (g-C_3_N_4_) to form a suspension. g-C_3_N_4_ has poor conductivity but a high electron-hole recombination rate that creates catalytically active sites [71]. The resulting suspension was drop-casted onto a GCE, and the modified electrode retained 92% of its initial current response to 25 nM of CAP after being stored for 30 days at 4 °C. Interferents including various organic molecules, metal ions, and antibiotics were used at concentrations ranging from 10 to 500 times greater than CAP, but they had no impact on the CAP signal. Another team used WO_4_ to form zinc tungstate (ZnWO_4_), which has high chemical stability, catalytic activity, and electronic versatility [72]. The ZnWO_4_ suspension was drop-casted onto a GCE, and only clindamycin and thiamphenicol were used as interferents, but they had no impact on the CAP signal.

One team created a composite using silver nanowires (AgNWs) and carbon nanotubes (CNTs), which was drop-casted onto a GCE [73]. When assessed for selectivity, metronidazole at a concentration 20 times greater than CAP increased the CAP peak from 17.8 to 93.8 µA. Bimetallic nanoparticles like Pt-Pd enhance electrochemical properties due to the coupling of two strong metals [74]. Pt-Pd was combined with rGO to form a composite, which can form strong Van der Waals interactions and improve stability and conductivity. The resulting Pt-Pd/rGO suspension was drop-casted onto a GCE, and the modified electrode retained 90.6% of the initial current response. Interferents, including glucose, ascorbic acid, and chlortetracycline, were used to assess for selectivity, and the peak change was less than 5%. Another team developed a composite consisting of rGO, MWCNTs, and CB, integrated with MoS_2_, a material known for its excellent catalytic activity. This composite was drop-casted onto a GCE [75]. Although this electrode exhibited a higher LOD compared to the previously reported system, its full potential remains uncertain, as it was neither evaluated in real samples nor tested against common interferents.

**Figure 8:**
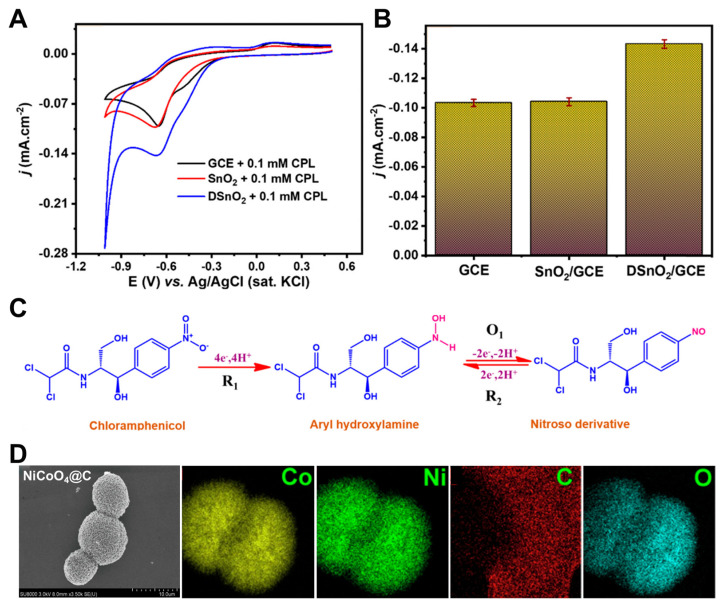
(**A**) CV curves and (**B**) comparative catalytic activity of bare GCE, SnO_2_/GCE, and DSnO_2_/GCE. (**C**) Proposed sensing mechanism for CAP detection. Adapted with permission from Ref. [76]. Copyright 2025 Royal Society of Chemistry. (**D**) SEM image and corresponding EDS elemental mappings of NiCoO_4_@C nanoparticles. Adapted with permission from Ref. [77]. Copyright 2021 Elsevier.

Tin oxide (SnO_2_) is a chemically stable, non-toxic, and intrinsically n-type semiconductor characterized by a high isoelectric point, excellent electrical conductivity, and an extensive electrochemically active surface area. These attributes render SnO_2_ highly attractive for electrochemical sensing platforms. Nevertheless, its practical utility is often constrained by sluggish interfacial electron transfer and suboptimal catalytic efficiency. To address these limitations, oxygen-deficient tin oxide (DSnO_2_) nanoparticles were rationally engineered to introduce a high density of oxygen vacancies, which serve as active sites for electron exchange and significantly enhance charge carrier density [76]. The presence of these structural defects in DSnO_2_ facilitates rapid electron transport and improves interfacial kinetics at the electrode–electrolyte boundary, thereby elevating the overall electrochemical performance (Figure 8A,B). The synthesized DSnO_2_ nanoparticles were incorporated into a nanocomposite matrix and subsequently immobilized onto a GCE via a drop-casting method, forming a stable and highly responsive electrochemical sensing interface. The modified GCE exhibited markedly improved sensitivity and selectivity toward CAP detection, with the proposed sensing mechanism illustrated in Figure 8C. This enhanced performance is attributed to the synergistic effects between the oxygen vacancies and the conductive network within the nanocomposite. Such synergy not only promotes efficient redox processes but also minimizes interfacial charge-transfer resistance, offering a promising strategy for high-performance antibiotic sensing.

Binary spinel structures are a class of crystalline materials known for their enhanced electrochemical activity, primarily arising from the synergistic interaction between two bimetallic ions. However, traditional spinels often suffer from limited electrical conductivity and moderate structural stability. To overcome these limitations, nickel (Ni) and cobalt (Co) were selected to form a nickel cobaltite (NiCo_2_O_4_) spinel, leveraging their multiple oxidation states and abundant valence electrons to facilitate rapid electron transfer and redox cycling [77]. The resulting nickel cobaltite with a carbon NiCo_2_O_4_@C composite exhibited a highly porous nanostructure, which provided many electrochemically active sites, thereby enhancing catalytic performance (Figure 8D). The prepared suspension was drop-casted onto a GCE, and after one week of storage, the sensor demonstrated excellent signal retention, with only a 4.41% decrease in the CAP peak current. Moreover, this sensor was capable of simultaneously detecting CAP and furazolidone, demonstrating its dual analyte sensing capability and potential applicability in complex sample matrices.

Europium oxide (Eu_2_O_3_) is a rare-earth metal oxide known for its excellent electrical conductivity and redox properties. However, its intrinsic sensing performance is limited by moderate chemical stability and relatively low surface area, which restricts its interaction with target analytes [78]. To overcome these limitations, a Eu_2_O_3_/rGO composite was fabricated by combining Eu_2_O_3_ nanoparticles with rGO sheets, which offer a high specific surface area, abundant π-π conjugated domains, and rapid electron transport pathways. The synergistic interaction between Eu_2_O_3_ and rGO not only enhances the electron transfer kinetics at the electrode–electrolyte interface but also promotes stronger adsorption of analyte molecules such as CAP through π–π stacking and possible coordination with Eu^3+^ centers. This modified electrode exhibited excellent sensitivity toward CAP even in the presence of common interfering substances, including glucose, 4-nitrobenzene, 4-nitrophenol, 4-aminophenol, and 4-nitroaniline, indicating high selectivity. Furthermore, the sensor demonstrated good storage stability, retaining approximately 96.8% of its initial current response after being stored at 5 °C for 10 days, highlighting its potential for long-term environmental monitoring applications. Table 4 provides an overview of representative hybrid-based electrochemical sensors for CAP detection.

## 4. Challenges in CAP Detection

### 4.1. Instability of Sensing Materials

Instability poses a major challenge in the use of various sensing materials, particularly nanomaterials for sensing applications. Several factors contribute to this issue, including environmental conditions (air, humidity, and light), mechanical stress, and the intrinsic properties of the nanomaterial. Such instability can alter surface chemistry or even induce structural changes, which directly impact the reproducibility, accuracy, and reliability of sensor performance. Transition metals, for example, silver and copper nanomaterials, oxidize rapidly in air due to their high surface reactivity, unlike their bulk counterparts [51,73]. Some transition metal oxides and dichalcogenides can undergo changes in their properties under ambient air and humidity. For example, CuO nanomaterials suffer from surface redox transitions (Cu^2+^ ↔ Cu^+^) and potential reduction to Cu_2_O, making them moderately unstable under ambient conditions [69]. MoS_2_, nanomaterials, particularly in few-layer or monolayer form, are even more vulnerable, as they readily oxidize in air and light to form sulfates, which severely limits long-term stability [75].

### 4.2. Matrix-Induced Electrochemical Interference

Beyond the presence of humic substances and suspended solids, CAP detection in complex samples is hindered by electroactive co-contaminants such as phenolic compounds, nitroaromatics, and redox-active metal ions, which can generate overlapping oxidation/reduction peaks in voltammetric measurements [79]. These interferences often mask or distort CAP signals, particularly in the sensitivity analysis of DPV or SWV. Advanced signal deconvolution methods, such as multivariate curve resolution–alternating least squares (MCR-ALS) and machine-learning-based peak discrimination, have shown promise in resolving overlapping responses but remain underutilized in current CAP sensing research [80,81].

### 4.3. Surface Fouling and Biofilm Formation

During environmental monitoring, electrode surfaces are often subjected to fouling due to the adsorption of organic macromolecules, including proteins and polysaccharides, or due to the formation of microbial biofilms [82]. This fouling process can gradually reduce sensor sensitivity and cause baseline drift. Antifouling strategies such as the use of zwitterionic polymers, polyethylene glycol (PEG) modification, or patterned hydrophilic-hydrophobic surfaces have been proposed in biosensing, but their long-term effectiveness in detection, particularly under continuous exposure in the field, has not been systematically examined [83].

### 4.4. Lack of Harmonized Validation Protocols

Validation procedures for CAP sensors vary widely among studies. Differences in calibration matrices, spiking concentrations, and recovery calculation methods hinder a direct comparison of analytical performance and slow the establishment of benchmark standards. Adopting internationally harmonized validation protocols, like the EU Commission guidelines for pesticide residue analysis, would facilitate regulatory acceptance and promote technology transfer to certified testing laboratories [84,85].

### 4.5. Operational Stability Under Variable Environmental Conditions

While storage stability is important, operational stability during real-time deployment is equally critical. CAP reduction potentials are sensitive to pH changes due to proton-coupled electron transfer mechanisms, and this can affect the reproducibility of quantitative measurements in samples with fluctuating acidity [29]. Temperature variations can also degrade nanomaterial-based electrodes or cause drift in reference electrode potential [82]. Developing sensing platforms with built-in pH and temperature compensation, or integrating simultaneous environmental parameter monitoring, is essential for accurate detection in diverse field conditions [20].

### 4.6. Data Integration and Field Deployment

Even when electrochemical sensors achieve an adequate analytical performance, their practical deployment in the field is often limited by a lack of integrated systems for automated data collection and analysis. Combining CAP sensors with Internet of Things (IoT) architectures, cloud-based analytical platforms, and geospatial mapping could allow real-time contamination tracking and inform decision making in environmental management [21,86]. These systems require reliable wireless communication, efficient power management, and secure data handling, all of which remain underdeveloped in current CAP detection studies.

## 5. Future Perspectives

Despite significant advances in the development of electrochemical sensors for CAP detection, several critical challenges must still be addressed to enable practical deployment in environmental and food safety applications. While numerous studies have reported low limits of detection, high sensitivity, and acceptable selectivity using nanomaterial-based electrodes such as metal oxides, bimetallic spinels, doped semiconductors, and carbon-based hybrids, many of these platforms remain limited in terms of real-world utility.

*Selectivity*—Assessments in several reports remain superficial, often limited to common inorganic ions or structurally unrelated molecules, while structurally analogous antibiotics or biochemically relevant interferents are rarely included. Given the widespread misuse of CAP and its co-occurrence with other antibiotics and organic pollutants in environmental matrices, future sensor systems must be rigorously evaluated against chemically similar compounds to ensure analytical specificity.

*Stability*—There have been several electrodes that have shown significant signal degradation after short-term storage under ambient conditions. For instance, some sensors retained less than 85% of their initial response after 10–20 days, which is insufficient for practical field use. Although promising results have been achieved, such as the composites of SrMoO_4_–SGCN and Eu_2_O_3_/rGO, these remain exceptions rather than the rule. Long-term electrochemical stability under variable environmental conditions should be a key focus in future studies.

*Scalability and Reproducibility*—While numerous laboratory-scale demonstrations of electrochemical sensors for CAP detection have achieved an impressive analytical performance, translating these platforms into large-scale, commercially viable products remains a major challenge. Inconsistencies in nanomaterial synthesis, electrode modification, and assembly can result in significant batch-to-batch variability, undermining reproducibility. Standardized manufacturing approaches such as screen printing, roll-to-roll nanomaterial deposition, and additive manufacturing (e.g., 3D printing) have shown potential for improving scalability while maintaining performance consistency. In parallel, the environmental footprint of sensor fabrication is becoming increasingly relevant in the context of global sustainability goals and the United Nations (UN) Sustainable Development Goals (SDGs). Integrating life cycle assessment (LCA) into the early design phase allows for the systematic evaluation of environmental impacts across raw material extraction, nanomaterial synthesis, device fabrication, operational energy consumption, and end-of-life disposal.

*Real Sample Validation*—Several advanced sensors with excellent laboratory performance (e.g., Co_3_O_4_@rGO, Cu-based nanocomposites, and MoS_2_ hybrids) were not tested in real matrices such as contaminated water, soil, or animal-derived food products, where complex interferences and matrix effects are inevitable. Moreover, some sensors were validated only in biological fluids like urine or eye drops, which do not align with the primary risk pathways of CAP exposure. Future research should prioritize validation in agriculturally and environmentally relevant samples (e.g., milk, poultry, and aquaculture wastewater) to enhance the translational value.

*Multi-Analyte Detection Platforms*—It represents a forward-looking direction in sensor development. Future electrochemical sensors should be designed to enable broad-spectrum, multiplexed detection of multiple contaminants while maintaining high sensitivity and specificity. Such contaminants may include other veterinary antibiotics frequently found in co-contaminated environments, such as tetracycline, kanamycin, sulfonamide, and florfenicol, as well as non-antibiotic pollutants like heavy metals (e.g., lead and mercury) and pesticide residues. This capability is particularly important given the frequent co-contamination of water and food sources with diverse hazardous compounds.

*Regulatory Applicability*—The successful integration of electrochemical sensors for CAP detection into regulatory frameworks is paramount for their practical deployment in food safety and environmental monitoring. Leading authorities such as the European Food Safety Authority (EFSA), U.S. Food and Drug Administration (FDA), and Codex Alimentarius Commission (CAC) enforce stringent maximum residue limits and zero-tolerance policies for CAP, reflecting its high toxicity and prohibition in food-producing animals. For instance, the EU’s Commission regulation mandates detection limits often below sub-µg/kg levels, while the FDA maintains an absolute zero tolerance for any detectable residues.

## 6. Conclusions

Metal- and carbon-modified electrodes have demonstrated significant potential for the sensitive and selective electrochemical detection of CAP. Among them, GEs remain a leading choice due to their outstanding electrical conductivity, chemical stability, and biocompatibility, which enable effective signal amplification and robust sensor platforms. The functionalization of these electrodes with aptamers—short, single-stranded DNA or RNA sequences—significantly enhances detection specificity through the selective molecular recognition of CAP. Further improvements have been achieved through the integration of bimetallic nanomaterials containing noble metal nanoparticles, which enhance electron transfer kinetics and catalytic activity. In addition, the application of advanced materials such as Gr derivatives, MOFs, COFs, and MIPs has markedly increased both the sensitivity and selectivity of CAP. Despite these advances, several challenges must be addressed before these technologies can be widely implemented in real-world settings. These include limited long-term operational stability, insufficient resistance to complex environmental interferents, and the lack of rigorous validation using real sample matrices. To overcome these limitations, future research should focus on the development of multi-analyte sensing platforms capable of simultaneously detecting multiple veterinary antibiotics, reflecting the frequent co-occurrence of drug residues in food and environmental samples. Additionally, the exploration of novel doped nanomaterials, biocompatible composites, and hybrid nanostructures may offer enhanced catalytic efficiency and structural robustness. The successful integration of these materials into scalable, flexible, and cost-effective electrode designs will be critical for developing next-generation electrochemical sensors that are not only highly reliable but also suitable for PoC, field, and regulatory applications.

## Figures and Tables

**Table 1:** Toxicological mechanisms and chemical basis of CAP in humans.

Symptom	Physiological Basis	Refs.
Bone marrow toxicity	CAP targets 70S-like mitochondrial ribosomes in hematopoietic progenitor cells, inhibiting mitochondrial protein synthesis and impairing hematopoiesis.	[7,8]
Reversible anemia	Dose-dependent mitochondrial dysfunction in erythroid precursors transiently suppresses red blood cell production, which typically normalizes after drug withdrawal.	[8]
Blood dyscrasia	Reactive CAP metabolites and oxidative stress damage rapidly dividing bone marrow cells, disrupting hematopoiesis across multiple lineages, including leukocytes and platelets.	[9,12]
Hepatotoxicity	Impaired hepatic glucuronidation leads to the accumulation of CAP unmetabolized and reactive nitrometabolite intermediates, causing oxidative liver injury and potential cytotoxicity.	[10,13]
Neurotoxicity	CAP crosses the blood–brain barrier due to its high lipophilicity and disrupts mitochondrial respiration in neurons, potentially resulting in optic or peripheral neuropathy.	[10,14]
Gray baby syndrome	In neonates, immature hepatic and renal systems prevent proper metabolism and excretion of CAP, leading to systemic accumulation, cardiovascular collapse, and lactic acidosis.	[10,11]
Aplastic anemia	A rare, idiosyncratic condition possibly triggered by genotoxic nitroso metabolites or immune-mediated mechanisms, causing irreversible bone marrow failure and pancytopenia.	[8,10,11]

**Table 2:** Metal-based electrochemical sensors for CAP detection.

Functional Electrodes	Methods	Linear Range (nM)	LOD (nM)	Real Samples	Ref.
Aptamer/PDDA-Gr/PtPd@Ni-Co/GE	DPV	1 × 10^–5^–10	9.85 × 10^–7^	Honey	[30]
Aptamer/UiO-66-NH_2_@COF/GE	EIS	3.1 × 10^–5^–15.5	2 × 10^–5^	Milk, human serum, river water, urine	[31]
Aptamer/Au@COF/GO-NH_2_/GE	EIS	1.55 × 10^–4^–3.09	4.99 × 10^–5^	Milk, human serum, river water	[32]
Aptamer/DNA/GE	DPV	1–1000	0.29	Honey	[33]
MIP-PANI/GE	DPV	10–1.0 × 10^6^	1.24	N/A	[34]
MIP-P(*o*-PD)/Pt	SWV	0.9–10	0.39	Honey, milk	[38]
Pt	DPV	2.48 × 10^3^–9.29 × 10^4^	N/A	Milk	[39]
AgCl/MoS_2_/ITO	Amperometry	4 × 10^3^–5.31 × 10^5^	1.93 × 10^3^	Honey, milk	[36]

**Table 3:** Carbon-based electrochemical sensors for CAP detection.

Functional Electrodes	Methods	Linear Range (nM)	LOD (nM)	Real Samples	Ref.
Aptamer/AgNPs/[NH_2_-Si]-GO/GCE	DPV	0.01–200	3.3 × 10^–3^	Honey, milk	[40]
Aptamer/AgNPs/3-ampy-RGO/GCE	EIS	1 × 10^–3^–1	3 × 10^–4^	Milk	[41]
Aptamer/MOF/MGNP/GCE	SWV	1 × 10^–4^–50	3.3 × 10^–5^	Milk	[42]
Aptamer-MIP/AuNPs/CS-MWCNT/GCE	DPV	0.031–3.1 × 10^4^	0.01	Honey, milk, sewage	[43]
Au/rGO/SPCE	DPV	50–1 × 10^5^	1	Poultry feed, human serum, honey, milk, egg	[44]
Au/SPCE	CV, DPV	250–5 × 10^4^	100	N/A	[45]
AuNPs/BDD	SWV	5 × 10^3^–3.5 × 10^4^	5 × 10^3^	N/A	[46]
AuNPs/GO/GCE	Amperometry	1.5 × 10^3^–2.95 × 10^3^	250	Honey, milk, powdered milk, eye drops	[47]
Au/N-Gr/GCE	LSV	2 × 10^3^–8 × 10^4^	590	Eye drops	[48]
Aptamer/GA/p(1,5-DAN)/EPPG	SWV	5 × 10^–5^–5 × 10^–4^	1.1 × 10^–5^	Rosetta cell, Mycin, Paraxin-250	[49]
Aptamer/p-AHNSA/EPPG	SWV	0.1–2.5 × 10^3^	0.02	Paraxin-250, Chloromycetin	[50]
CuNDs/MWCNTs/GCE	LSV	150–1.2 × 10^4^	9.84	Lake water	[51]
MWCNTs@MIP/CMK-3/P-r-Gr/GCE	DPV	5–4 × 10^3^	0.1	Honey, milk	[52]
MIP/PANI/AuNPs-rGO-CS/GCE	SWV	1–200	0.063	Milk, egg	[53]
SWCNHs-COOH/GCE	LSV	100–1 × 10^5^	100	Lake water	[54]
3D-rGO/GCE	DPV	1 × 10^3^–3.3 × 10^5^	150	Milk, eye drops	[55]
rGO/GCE	DPV	1 × 10^4^–6.6 × 10^4^	220	Milk	[56]
Cl-rGO/GCE	DPV	2 × 10^3^–3.5 × 10^4^	1 × 10^3^	Milk, eye drops, calf plasma, tap water	[57]
Sn/rGO/SPCE	DPV	500–1 × 10^5^	200	Honey, milk, eye drops	[29]
GNFs/GCE	Amperometry, DPV	0.5–5.5, 10–270	0.38, 4.4	Urine	[58]
β-CD/CMK-3@PDA/GCE	SWV	500–5 × 10^5^	200	Powdered milk, bee pollen	[59]
PLA/CB	DPV	1 × 10^4^–3.31 × 10^5^	980	Milk, tap water	[60]
PGE	LSV, SWV	2.5 × 10^3^–1 × 10^6^, 2.5 × 10^3^–7.5 × 10^5^	1.39 × 10^3^, 609	Pharmaceutical capsules	[61]

**Table 4:** Hybrid-based electrochemical sensors for CAP detection.

Functional Electrodes	Methods	Linear Range (nM)	LOD (nM)	Real Samples	Ref.
Fe_3_O_4_/CFME	DPV	400–1 × 10^3^	167	Sediment	[62]
Fe_3_O_4_/GCE	SWV	90–4.7 × 10^4^	90	Shrimp	[63]
Co-Fe_3_O_4_ NS/GO/GCE	DPV	5–1.52 × 10^5^	1.04	Milk	[64]
Ho^3+^/Co_3_O_4_/GCE	DPV	10–8 × 10^5^	7.1	Human serum, eye drops, urine	[66]
Co_3_O_4_@rGO/GCE	Amperometry, CV, DPV	100–1.5 × 10^6^, 1 × 10^3^–2 × 10^6^, 2 × 10^3^–2 × 10^6^	100, 550, 1.16 × 10^3^	Honey, milk	[67]
SrMoO_4_/SGCN/GCE	Amperometry	5–1.316 × 10^6^	1.5	Human serum, river water, urine	[65]
Sr-ZnO@rGO/SPCE	LSV	190–2.847 × 10^6^	130	Milk, powdered milk	[68]
Cu/SPCE	DPV	2.5 × 10^3^–5 × 10^4^	250	Honey, milk	[69]
CuO/SPCE		1 × 10^3^–5 × 10^4^	450	N/A	
Cu_2_O/SPCE		1 × 10^3^–5 × 10^4^	230	Honey, milk	
Cu-MoS_2_/SPCE		500–5 × 10^4^	190	Honey, milk	
CCO/SPCE	DPV	2.5 × 10^3^–5 × 10^4^	660	Milk	[70]
CCO-GO/SPCE		2.5 × 10^3^–5 × 10^4^	710	Milk	
CFO/SPCE		2.5 × 10^3^–5 × 10^4^	250	Milk	
CFO-GO/SPCE		2.5 × 10^3^–5 × 10^4^	410	Milk	
g-C_3_N_4_/MnWO_4_/GCE	DPV	4–71	1.03	Blood serum, milk, sewage, river water	[71]
ZnWO_4_/GCE	CV	5 × 10^4^–5 × 10^5^	320	N/A	[72]
CNTs/AgNWs/GCE	DPV	100–1 × 10^5^	80	River water, tap water	[73]
Pt-Pd/rGO/GCE	LSV	200–3 × 10^4^	100	Milk	[74]
MoS_2_-rGO/GCE	DPV	5 × 10^3^–3.5 × 10^4^	1 × 10^3^	N/A	[75]
MoS_2_-MWCNTs/GCE		1 × 10^3^–3.5 × 10^4^	400	N/A	
MoS_2_-CB/GCE		5 × 10^3^–5.5 × 10^4^	1.9 × 10^3^	N/A	
DSnO_2_/GCE	DPV	100–3.5 × 10^4^	94	Honey, milk, tap water	[76]
NiCo_2_O_4_@C/GCE	DPV	500–4.3 × 10^5^	44	Honey, milk	[77]
Eu_2_O_3_/rGO/SPCE	Amperometry	20–800 × 10^3^	1.32	Honey, milk	[78]

## Data Availability

Data are contained within the article.

## Abbreviations

3-ampy-RGO	3-aminomethyl pyridine-functionalized graphene oxide
3D-rGO	three-dimensional reduced graphene oxide
AFM	atomic force microscopy
AB_2_O_4_	spinel oxides
AgCl	silver chloride
AgNP	silver nanoparticle
AMR	antimicrobial resistance
AuNP	gold nanoparticle
BDD	boron-doped diamond
CAC	Codex Alimentarius Commission
CAP	chloramphenicol
CB	carbon black
CCO	copper cobaltite
CFME	carbon fiber microelectrode
CFO	copper ferrite
Cl-rGO	chlorine-doped reduced graphene oxide
CMK-3	ordered mesoporous carbon
CNT	carbon nanotubes
Co_3_O_4_	cobalt oxide
Co	cobalt
Cu	copper
Cu_2_O	cuprous oxide
CuO	cupric oxide
COF	covalent organic framework
Co-Fe_3_O_4_ NS/GO	cobalt-doped Fe_3_O_4_ nanospheres deposited on graphene oxide
CS	chitosan
CS-MWCNT	chitosan-functionalized multi-walled carbon nanotube
CuND	nanodendrite
CV	cyclic voltammetry
DPV	differential pulse voltammetry
EDS	energy-dispersive X-ray spectroscopy
EUCAST	European Committee on Antimicrobial Susceptibility Testing
EFSA	European Food Safety Authority
ELISA	enzyme-linked immunosorbent assay
EPPG	edge plane pyrolytic graphite
EU	European Union
Eu_2_O_3_	europium oxide
FDA	U.S. Food and Drug Administration
Fe_3_O_4_	iron oxide
GCE	glassy carbon electrode
GCN	graphitic carbon nitride
GE	gold electrode
GNF	graphene nanoflakes
GO	graphene oxide
Gr	graphene
Ho	holmium
IoT	Internet of Things
ITO	indium tin oxide
LCA	life cycle assessment
LOD	limit of detection
LSV	linear sweep voltammetry
MGNP	magnetic gold nanoparticle
MIP	molecular imprinted polymer
MnWO_4_	manganese tungstate
MOF	metal–organic framework
MoS_2_	molybdenum disulfide
MWCNT	multi-walled carbon nanotube
N-Gr	nitrogen-doped graphene
NH_2_-Si	triethoxysilane
Ni	nickel
Ni-Co	nickel–cobalt
NiCo_2_O_4_	nickel cobaltite
NW	nanowire
p(1,5-DAN)	poly(1,5-diaminonapthalene)
P(*o*-PD)	poly(*o*-phenylenediamine)
p-AHNSA	poly-(4-amino-3-hydroxynaphthalene sulfonic acid
PANI	polyaniline
PDA	polydopamine
PDDA-Gr	graphene functionalized poly(diallyldimethylammonium chloride)
PEG	polyethylene glycol
PGE	pencil graphite electrode
PLA	polylactic acid
P-r-Gr	three-dimensional porous graphene
Pt	platinum
Pt-Pd	platinum–palladium
rGO	reduced graphene oxide
SDGs	Sustainable Development Goals
SELEX	systematic evolution of ligands by exponential enrichment
SEM	scanning electron microscopy
SGCN	sulfur doped-graphitic carbon nitride
Sn/rGO	tin/reduced graphene oxide
SPCE	screen-printed carbon electrode
SrMoO_4_	strontium molybdate
SWCNH	single-walled carbon nanohorn
SWV	square-wave voltammetry
Sr	strontium
Sr-ZnO	strontium doped zinc oxide
TEM	transmission electron microscopy
UN	United Nations
US	United States
ZnO	zinc oxide
ZnWO_4_	zinc tungstate
β-CD	β-cyclodextrin
